# Specialties in Dental Practice

**Published:** 1891-10

**Authors:** 


### Editorial.

### Specialties in Dental Practice.

Dentistry as a specialty of medicine has been much discussed during the last few years, with the result of pretty well settling the relationship between dentistry and medicine. It is now about as generally conceded to be a department of medical practice, using this term in its broadest sense, as ophthalmology, laryngology or gynæcology: but it is not this specialization that we will consider here, but it is that of separating dental practice into specialties or departments. We remember well that, years ago, occasionally persons would give as a reason for not engaging in dental study and practice, that there was not enough in it, was not a sufficiently broad field to occupy the time, attention and energy of the man of high aspirations.

Such a declaration, however, even in former times, was an indication of a narrow, ignorant mind that possessed no adequate understanding of the subject in hand. There have been for many years some instances of specialization in dental practice. The very best practitioners of prosthetic dentistry in the profession were those who had devoted themselves exclusively to that line; such cases, however, have been confined mainly to the larger cities; but what has been done there, in a few instances, gives encouragement for hope that on a much broader scale the practice could be carried out to the great advantage of both the profession and the public, who are to be served.

The most perfect continuous gum work that has ever been produced was the result of those who devoted almost their entire time and attention to it; as illustration of this reference need only to be made to such men as John Allen, L. P. Haskell and Ambler Tees. The finest metallic plate work is only produced by those who give a large share of their time, attention and effort to it. The same principle is well illustrated by our finest crown and bridge workers. The finest operators in filling teeth are those who devote themselves most assiduously to it, and give most other departments the "go by."

The proper and most efficient medical treatment for diseases of the mouth, require a skill and thoroughness that is very difficult, if not impossible, to attain by one who attempts to follow up in daily practice each of the particulars composing it. It will hardly be affirmed by any one, that the man of general practice, is as thorough and efficient, as he who centers his efforts upon one or two particulars. It has been said, "that the specialist is always a narrow man." In one sense that may be true, but such narrowness always implies greater penetration and depth; and this in many things is preferable to being thinly spread out over a large surface. While the efforts of the specialist may be exercised within a narrow sphere, it by no means follows that his mind is narrow. The most thorough and successful specialist reaches out in all directions for resources that may be made subservient to his particular line of thought and work; and these resources usually are made more definitely subservient to his purpose, than he who gathers up such resources merely for the purposes of using them in some way, and for some object that may occur to him at some indefinite time; indeed the attainment of knowledge and resources with some people, is little more than a "Toodles' door plate" affair. He who deals in specialties needs as broad culture and range of knowledge, as those who attempt to cultivate more territory.

The question very naturally follows here, what course of elementary training does the student require who proposes to follow a specialty for his life work? It can hardly be denied, that the broadest culture should be encouraged, because of the training it gives, and preparation of the mind and hand for future work, and for the accumulation of knowledge and facts, that may be in some way utilized in the accomplishment of special work. After this broad culture and general education is attained then let specialties receive attention; specialization so far as environments will permit. The ordinary course of dental education comes up to the point where specialization should begin, and usually from this point the specialist has been compelled in the main, to proceed alone, though he really needs assistance in the preparation for his particular work. This fact is being recognized in the establishment of Post Graduate schools or departments in dental colleges. Without doubt, however, this department of work should receive more attention than has as yet been given to it in connection with any dental college. The Post Graduate course, established by Dr. L. P. Haskell, of Chicago, comes nearer satisfying the demand in this direction, especially as far as prosthetic dentistry is concerned, than has anywhere else been realized. What has been done for prosthetic dentistry in this department, ought somewhere to be done for filling and treating teeth, for Oral Medicine and Surgery, for orthodontia, and perhaps for crown and bridge work. It would seem that this work could be done more economically in connection with dental colleges than by independent institutes. There will doubtless be a variety of opinions in regard to this question, as to how the most thorough work can be done; but our anxiety need not be so much exercised as to where it is done, as to the fact, that it be most thoroughly done. Let this question with all that pertains to it be in the minds, and receive the special attention of those in our profession, who are in any wise engaged in giving shape to its interest and its destiny. College men, Association men, and all who have the true interest of our profession at heart, think upon these things.

---

THE proceeding of the American Dental Association a portion of which was published in the September REGISTER, will be continued and completed in the November number. The report was not completed in time for this number.

